# Impact of gut microbiota and its metabolites on immunometabolism in colorectal cancer

**DOI:** 10.1097/IN9.0000000000000050

**Published:** 2024-11-28

**Authors:** Madison Flory, Paloma Bravo, Ashfaqul Alam

**Affiliations:** 1 Department of Microbiology, Immunology and Molecular Genetics, University of Kentucky, Lexington, KY, USA; 2 Department of Biology, Carleton College, Northfield, MN, USA; 3 Markey Cancer Center, University of Kentucky, Lexington, KY, USA

**Keywords:** gut microbiota, microbiome, metabolites, immunometabolism, colorectal cancer

## Abstract

Colorectal cancer (CRC) is highly prevalent, accounting for approximately one-tenth of cancer cases and deaths globally. It stands as the second most deadly and third most common cancer type. Although the gut microbiota has been implicated in CRC carcinogenesis for the last several decades, it remains one of the least understood risk factors for CRC development, as the gut microbiota is highly diverse and variable. Many studies have uncovered unique microbial signatures in CRC patients compared with healthy matched controls, with variations dependent on patient age, disease stage, and location. In addition, mechanistic studies revealed that tumor-associated bacteria produce diverse metabolites, proteins, and macromolecules during tumor development and progression in the colon, which impact both cancer cells and immune cells. Here, we summarize microbiota’s role in tumor development and progression, then we discuss how the metabolic alterations in CRC tumor cells, immune cells, and the tumor microenvironment result in the reprogramming of activation, differentiation, functions, and phenotypes of immune cells within the tumor. Tumor-associated microbiota also undergoes metabolic adaptation to survive within the tumor environment, leading to immune evasion, accumulation of mutations, and impairment of immune cells. Finally, we conclude with a discussion on the interplay between gut microbiota, immunometabolism, and CRC, highlighting a complex interaction that influences cancer development, progression, and cancer therapy efficacy.

## 1. Introduction

Typically, the human microbiota and our own intestinal cells exist in harmony. But what happens when that balance is disturbed? Dysbiosis contributes to a variety of health problems, perhaps the most insidious of which is cancer. The precise role of the microbiome in colorectal cancer (CRC) has long been debated, due to the array of unique microorganisms and their specific interactions and environments within the colonic space. There is a vast preponderance of evidence supporting the distinct roles of certain microbes in the development of cancers, for example, the long-standing association of *Helicobacter pylori* infection with gastric cancer and the newer connections between CRC and *Bacteroides fragilis*, *Streptococcus gallolyticus, Fusobacterium nucleatum*, and certain types of *Escherichia coli* ^[1–7]^. However, the contributions of other bacterial species to cancer development are much more ambiguous. Although most of the information we currently have explores the roles of bacteria in CRC, the microbiome also contains innumerable viruses, fungi, and bacteriophages that almost certainly have effects on CRC as well. Due to this lack of information, this review will focus on the roles of bacteria in CRC and their effects on the immune system, but it is important to note this gap in our understanding of the complete effect of the microbiome on CRC. Data currently supports a variety of individual roles for individual bacteria in cancers, and only by untangling the complex web of microbial-microbial and microbial-host interactions, including interactions with the immune system, can we identify specific microbial contributions to CRC and uncover the mysterious roles of the microbiome in CRC.

## 2. Colorectal cancer

CRC is not only one of the world’s most prevalent types of cancer but also one of the deadliest types of cancer. Approximately one-tenth of both world’s cancer deaths and occurrences are due to CRC ^[8]^. The International Agency for Research on Cancer reports that 1.93 million new cases of CRC were diagnosed during 2020 and that CRC was a contributing factor in nearly one million deaths ^[8]^. Treatment consists primarily of surgery to remove the affected area of the intestine and chemotherapy, both of which can be debilitating. Additionally, these treatments become much less effective when applied to advanced-stage CRC ^[9]^. Screening and polyp removal through colonoscopy remains one of the most effective strategies for preventing advanced CRC ^[10]^. Although many advances are being made in the detection and treatment of CRC, and thus overall death rates have stagnated in some areas, CRC cases are still generally increasing across the world, especially in younger populations ^[11]^. The development of CRC can be attributed to a complex constellation of risk factors, including inflammatory bowel disease (IBD), alcohol use, diet, genetics, smoking, obesity, and dysbiosis of the microbiome, that all collide to provide a carefully calibrated risk status for CRC development.

Although the gut microbiota has been implicated in CRC carcinogenesis for the last several decades, it remains one of the least understood risk factors for CRC development, as the gut microbiota is extremely diverse and variable. The gut microbiota composition is affected by many factors, including diet and infections, and even minor changes in bacterial populations, functions, or interactions can result in critical impacts on the microbiota’s effects on CRC development and progression. The microbiota directly affects host cell processes, including cell metabolism, proliferation, and survival, as well as overall host inflammation and immunity. These interactions are necessary for typical bodily functions, as the microbiome produces metabolic products used by the body for vital processes. Other interactions can cause direct harm to human cells, but even typically beneficial interactions can cause damage if they occur at too high of a rate. For this reason, microbiota-host interactions must exist in a meticulously controlled equilibrium to promote their beneficial effects for the host while minimizing any damage caused by their close interactions.

## 3. Immunometabolism

Metabolism and immunity are closely intertwined and pivotal in nutrient digestion, distribution, energy production, molecular building block synthesis, and tissue homeostasis, as well as in host defense. Over the past 20 years, research studies have unveiled the intricate relationship between metabolic dysregulation and immune dysfunction, particularly in metabolic diseases and cancer ^[12]^. Immunometabolic studies highlight how metabolic pathways such as glycolysis, oxidative phosphorylation, and amino acid metabolism impact systemic immunity, including cell function and activation ^[13]^. In addition, immunometabolism has emerged as a critical mechanism that governs adaptive and innate immune regulation and interweaves metabolic processes with immune responses.

Early studies revealed that many intrinsic and extrinsic factors stimulate inflammatory cytokines in obese adipose tissue, highlighting a link between metabolism and the immunological state. In addition, metabolic reprogramming, described by Warburg in cancer cells, is now recognized as a hallmark of immune cell activation ^[13]^. Cancer cells demonstrate sustained metabolic reprogramming, which mediates immune escape and drug resistance. Unlike tumor cells, immune cells in the tumor microenvironment may exhibit a bias toward an immunosuppressive state, eventuating in malignancy. A number of factors impact metabolic reprogramming, including oncogenes, mutated enzymes, oxygen availability, extracellular pH, and altered nutrients and energy sources. Thus, cancer immunometabolism explores how dysregulated metabolism in tumor cells influences immune cell function within the tumor microenvironment during tumorigenesis and metastasis ^[14]^. Cancer immunometabolism underscores how metabolic reprogramming in cancer cells, such as immune cell metabolic circuits and the Warburg effect, alters nutrient availability and antitumor immune responses by T cells, macrophages, and other immune cells ^[15]^. Although the metabolic hallmarks of cancer have been extensively studied, the metabolic states of immune cells have not been comprehensively examined for their effector functions, differentiation, and polarization.

The gut microbiome, which includes bacteria, yeast, fungi, and viruses, coexists with the host in a complex but balanced ecosystem. However, gut microbial dysbiosis, which is characterized by pathological alterations in microbial community profiles, contributes to low-grade inflammation and the pathophysiology of metabolic disorders, including obesity and diabetes ^[16]^. Similarly, microbiome profiling of stool samples collected from CRC patients also exhibits microbial dysbiosis in the intestine. Although the dysbiotic gut microbiome has been extensively studied, there is still a vacuum of knowledge regarding how the dysbiotic gut microbiota impacts immunometabolism in a pro-oncogenic manner ^[17]^. Emerging evidence suggests that the tumor-associated microbiome, identified by metagenomic analysis of the tumor tissues, modulates cancer development, immune suppression, metabolic reprogramming, and therapy efficacy through distinct metabolic pathways.

Recent metagenomic and metabolomic studies demonstrated alterations in CRC microbial profiles and revealed that these alterations in gut microbial metagenomes are associated with an imbalance in diverse bacterial enzymes and protein expression, leading to altered production of numerous small molecules and metabolites, including amino acids and their derivatives, short-chain fatty acids, phenolic compounds, and bile acids ^[18,19]^ These studies demonstrated that microbiota is an active actor in regulating the CRC immunometabolic network ^[20]^. In animal models, ex vivo studies using CRC tumor tissues, and CRC patients show that the small molecules and metabolites produced by the tumor microbiome have the potential to rewire signaling pathways in cancer and immune cells, offering potential avenues for therapeutic intervention ^[21–23]^. In this review, we highlight how the dysbiotic microbial metabolome promotes inflammation and metabolic dysfunction, exacerbating the development and progression of CRC. Next, we discuss the salient studies that suggest that the tumor microbiome modulates cancer development and therapy efficacy through distinct metabolic pathways and metabolites. Furthermore, we discuss the potential application of bacteria as cancer drugs and the possibility of targeting microbial metabolism as a novel therapeutic intervention in cancer treatment and management. Finally, we highlight the significance of the tumor-associated microbiome in cancer immunotherapy and discuss strategies to modulate microbial communities within the tumor microenvironment.

## 4. Gut microbiome

The human host and microbial communities are in constant crosstalk which influences that symbiotic relationships, aiding in homeostasis and modulating immune function. While “microbiota” and “microbiome” are frequently used interchangeably, there are distinctions between the two terms. Microbiota refers to the microorganisms present in a specific environment, such as the skin and gut microbiota. On the other hand, the microbiome encompasses the entire genetic material from all microorganisms within an environment, comprising the microbial community and the structural components, metabolites, and environmental factors. The intestinal microbiota differs significantly, both in numbers and taxonomy, along the anatomical regions and microniches of the gastrointestinal (GI) tract. Nevertheless, across the entirety of the GI tract, the most common microbes belong to the phyla of Bacteroidetes, Firmicutes, Proteobacteria, and Actinobacteria, with Bacteroidetes and Firmicutes representing the overwhelming majority of bacteria in the healthy gut microbiome. However, there are hundreds of individual bacterial species in the gut microbiome, varying in representation based on physiologic and anatomic factors.

Not only is the microbiome itself tremendously heterogeneous, but it also is immensely dynamic. The microbiome changes over time with input from host immune interactions, dependent on diet and infections, age, and environment. Certain microbial states are associated, based on both microbial frequency and function, with various disease states, including those of CRC and IBD. The gut microbiome profoundly affects nearly every other body system, including the gut itself. For example, in addition to the inherent connections between the GI, endocrine, and immune systems, connections have been made between the microbiome and neurodegenerative diseases and the microbiome and psychiatric conditions in what termed the “gut-brain axis” ^[24,25]^. In the case of CRC, the tumor microbiome references the specific subset of the gut microbiome in and directly near colorectal tumors ^[26]^. The tumor microbiome can differ vastly from the surrounding gut microbiota, containing different microorganisms, producing different products, and affecting surrounding cells in unique ways to further drive tumor development and progression ^[27]^. These differences in the microbiota of CRC patients compared with healthy microbiota are essential for advancing screening and treatment protocols, as uniquely identifiable microbial states are associated with tumor location, risk stratification, and response to treatment.

## 5. Gut microbe alterations in colorectal cancer

**Figure 1.**
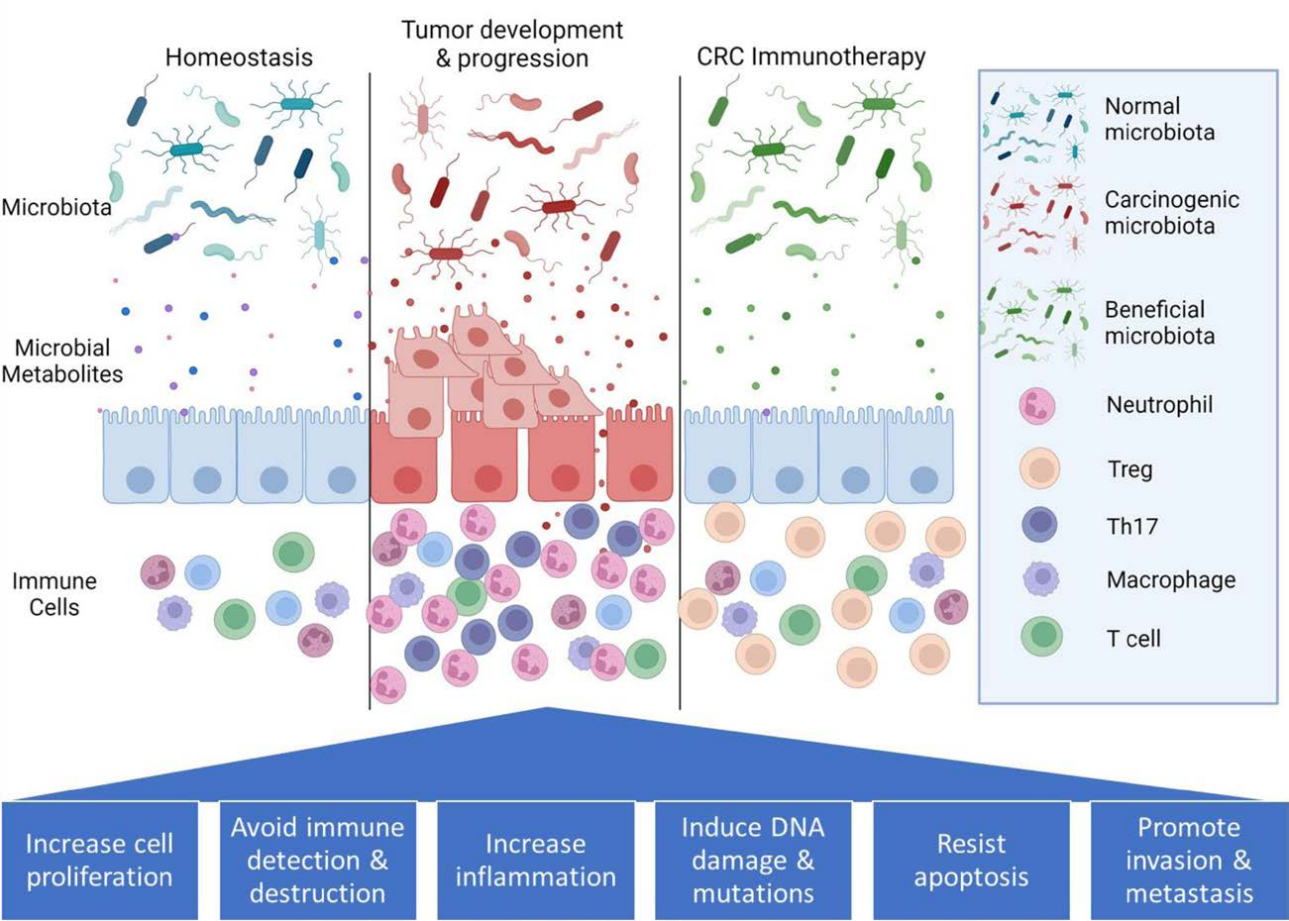
**Differential microbial states and their metabolites in the intestine have unique interactions with intestinal epithelial cells and immune cells.** Carcinogenic microbiota, including polyketide synthase + *Escherichia coli*, *Streptococcus gallolyticus*, and *Fusobacterium nucleatum*, are associated with an increase in inflammation, neutrophils, and a T_H_17 immune phenotype, along with several hallmarks of cancer. Beneficial microbiota, including *Akkermansia muciniphilia* and Lactobacillus species, produce a strong epithelial barrier and an increase in Treg cells. Figures created with BioRender.com (https://www.biorender.com/).

Many studies have noted unique microbial signatures in CRC patients compared to healthy matched controls, with variations dependent on patient age, disease stage, and location. Important to note, however, is that the correlation of certain species does not necessarily imply their direct involvement in CRC development or progression, and it remains unknown if the changes in the microbiome are the impetus for CRC development or if local and global microbiome modifications occur after the initiation of tumor formation (Figure 1). Overall decreases in microbial diversity were observed in CRC patient samples ^[28,29]^, but the diversity of microbial flora was higher in younger patients than in older ones ^[28]^. Specifically, increases in bacteria including *B. fragilis*, *F. nucleatum*, *Faecalibacterium prausnitzii*, *Akkermansia muciniphila*, and *E. coli* were observed in CRC cases ^[30]^. Differences in microbiomes can also be observed in healthy tissue, adenomas, and carcinomas, raising the question of stepwise changes versus unique microbial states for each stage of cancer development ^[31]^. Additionally, certain microbes are associated with differences in stage and location of CRC ^[32]^. Different tumor types showed distinct microbial phenotypes, with serrated and adenomatous polyps differing in Bacteroidetes and Proteobacteria populations ^[33]^. Decreases in potentially protective butyrate-secreting microbes were seen in tumor samples when compared with normal tissue ^[33]^. On a larger scale, the phyla of Bacteroidetes, Proteobacteria, and Fusobacteria have been seen to increase in CRC patients, while Firmicutes and Actinobacteria tend to decrease ^[34]^. The presence of biofilms has also been associated with colon adenomas and carcinomas, potentially contributing to a tumor-promoting microenvironment ^[35,36]^. These biofilms may be attributed to the invasion of the gut microbiome by bacteria from the oral microbiome, which are more commonly associated with biofilm formation. Increased levels of typically oral microbes have been found in CRC samples, including from the *Fusobacterium, Porphyromonas,* and *Peptostreptococcus* genera ^[31,37]^. Individual tumor samples from the same patient can also have unique microbiome microcommunities, including showing differences across a single tumor sample ^[38]^. Although similar changes in microbial diversity and form are common across CRC diagnoses, smaller differences attributed to each person’s individual characteristics remain, and it remains unknown to what degree these differences affect CRC development. Additionally, some studies exhibit conflicting findings, potentially due to differential study criteria, stratification or lack thereof by factors such as tumor location or stage, or inherent observed differences in microbial makeup by geography, for example ^[39,40]^. There also remains a gap to be filled in the identification of the association, mechanism of action, and roles for less-common microbes in the grand scheme of CRC development and progression.

## 6. Tumor microbiota

As a whole, the microbiome undoubtedly affects the development and progression of CRC. However, the subsection of the microbiota known as the tumor microbiome, which is closely associated with CRC tumors themselves, is a unique situation that may have even more of a dramatic impact on CRC. Microbial localization studies link certain microbes to tumors, while nearby unaffected intestinal epithelium does not play host to these microbes ^[41,42]^. Tumor microbiota can be extremely varied, differing even across multiple tumors in one individual ^[42]^. Many types of cancer are known to have a tumor microbiome, including brain, lung, bone, and breast cancer ^[26]^. In CRC, the tumor microbiome is known to differ based on the location of the tumor within the intestine; commonly, proximal tumors show increased diversity in comparison to distal colonic tumors, with one study identifying enrichment of more than 15 different phyla in proximal tumors compared with a single phylum in distal tumors ^[43]^. Interestingly, across individuals, tumor microbiomes tend to be more similar to each other than the microbes in unaffected areas, indicating that there may be a set of CRC intratumoral microbial signatures ^[44]^. These similarities and differences in tumor versus nontumor microbiota have implications for many aspects of an individual’s treatment and life after cancer, including the likelihood of response to various treatments, stage of cancer, and the likelihood of long-term survival. In one study, differences in tumor-specific microbiota were associated with a 7.5-fold difference in survival ^[45]^. Individual microbial signatures exist across various cancer classifications and may be due to the unique metabolic niches these bacteria find themselves in inside of tumors.

## 7. Tumor microbiota metabolism

Accumulating evidence suggests that many bacterially produced small molecules and metabolites, including short-chain fatty acids and bile acids, impact CRC development, progression, and treatment. These metabolites can have varying effects on tumor cells with multiple mechanisms of action. Some metabolites can induce DNA damage, which can contribute to the mutational burden of cancer cells, while others can encourage increased cellular proliferation, one of the many key hallmarks of cancer. Bile acids are one type of metabolite that many studies have found to play a role in CRC development. Bile acids are necessary metabolites that help to digest fats. They are classified as primary or secondary bile acids, with primary bile acids being directly derived from the liver, while secondary bile acids are created in the colon from the digestion of primary bile acids ^[46]^. Diet plays an important role in the amount of bile acids present in the body, as a high-fat diet will need more bile acids to digest the increased fat content. Extremely high levels of bile acids can cause direct membrane destruction of epithelial cells due to their status as cholesterol derivatives, destroying the epithelial barrier function and creating a proinflammatory environment ^[47]^. Increased levels of bile acids are connected to an increase in reactive oxygen species, causing oxidative stress to cells and inducing DNA damage ^[48]^. This DNA damage is global, but a high concentration of point mutations occurs in the K-ras gene, leading to activation of the mitogen activated protein kinase (MAPK) pathway and increased cell proliferation ^[48,49]^. Long-term exposure to bile acids is known to induce apoptosis resistance, an important aspect in the development of CRC. This occurs by proteolytic degradation of p53, the “guardian of the genome” ^[50]^. Bile acids, specifically deoxycholic acid, can also cause changes in several of the most common signaling pathways associated with cancer via tyrosine phosphorylation ^[51]^.

On the other hand, short-chain fatty acids, produced through fermentation in the gut, are associated with lowering the risk of CRC development. Short-chain fatty acids are known to induce an anti-inflammatory environment ^[52]^. Lower levels of short-chain fatty acids were seen in patients with CRC compared to patients without CRC ^[53]^. They are also known to inhibit histone deacetylases, which are typically upregulated in CRC, thus helping to change the way genes are expressed ^[52,54]^. Additionally, studies have examined the effect of short-chain fatty acids on the immune response in CRC. When CRC cells were exposed to short-chain fatty acids, they exhibited a more robust activation of CD8^+^ T cells, especially in CRC cells that had a high degree of microsatellite instability. In addition to higher activation of T cells, CRC cells that were exposed to short-chain fatty acids had more major histocompatibility complex class I present on their surfaces ^[55]^. Mowat et al showed that this effect occurred via GPCR41 signaling ^[55]^. Just these few examples of gut microbial metabolites show the wide variety of both metabolites and effects on colorectal tumors. The complex interplay between tumor cells, microbial metabolites, and the rest of the tumor microenvironment, including immune cells, leads to a specific and complex site-dependent situation that can have beneficial or negative effects on the development and progression of cancer.

In addition to changes in the numbers and diversity of microbial species represented in CRC, functional analyses of the microbiome have found differences in microbial metabolism. Meta-analysis found downregulation of carbohydrate metabolism by the microbiome in samples from CRC patients, and additionally, discovered enhancement of multiple amino acid, fatty acid, and glycoprotein catabolic pathways ^[56]^. Additional studies showed increases in nucleotide and amino acid biosynthesis ^[30]^. Analysis of samples from CRC patients also showed increases in DNA and protein binding, translation, DNA synthesis, and ribosome production pathways ^[28]^. Increases in bacterial metabolites implicated in the regulation of cell proliferation have also been found in CRC-associated biofilms ^[57,58]^. Butyrate, a short-chain fatty acid, and perhaps the most famous microbial metabolite implicated in CRC, slows cell proliferation and increases apoptosis, providing a benefit in the context of cancer ^[59]^. Other metabolites, however, may serve to increase cellular proliferation, increase the rate of mutation and DNA damage, or prevent apoptosis from occurring, promoting an intestinal state that is advantageous for the development of cancer ^[60]^. Thus far, we have focused on general differences in the microbiota itself and its metabolic products. Although each individual microbe may have an important role to play in the ultimate enterprise of CRC development, certain microbes have more established roles and mechanisms, and it is these we will primarily focus on moving forward.

## 8. Focus on specific bacteria

Building on the observed differences of specific microbial species in CRC has led to an exploration of specific roles for some of the most commonly associated CRC bacteria. Identifying the processes by which these bacteria act to induce or advance the progression of CRC may help to identify a stage at which this process can be interrupted, providing a potential intervention for CRC. In addition to interventions, we can also find potential future uses for the microbiome in CRC diagnosis, screening recommendations, and as predictive biomarkers for various aspects of CRC diagnosis, prognosis, and treatment. Some microbes may be protective against CRC, while many are neutral, and some are known to be detrimental. While many of the pathways and mechanisms through which microbes influence the development of CRC are unique, common outcomes from these bacteria result in increased intestinal inflammation, as well as creating an environment more conducive to tumor development, including increasing cellular proliferation, localized angiogenesis, higher rates of mutation and DNA damage, and decreased rates of apoptosis. Together, these effects combine to create a haven for tumor development, potentially due to the actions of one or many intestinal microbes. The mechanisms underlying these cancer-promoting processes can be direct or indirect. These differences may be due to interactions between the microbiome, intestinal epithelial cells, and immune cells. Here, we focus on some of the most highly supported bacteria and their mechanisms and impacts surrounding CRC, as well as noting the potential for future work.

### 8.1 Bacteroides fragilis

**Figure 2.**
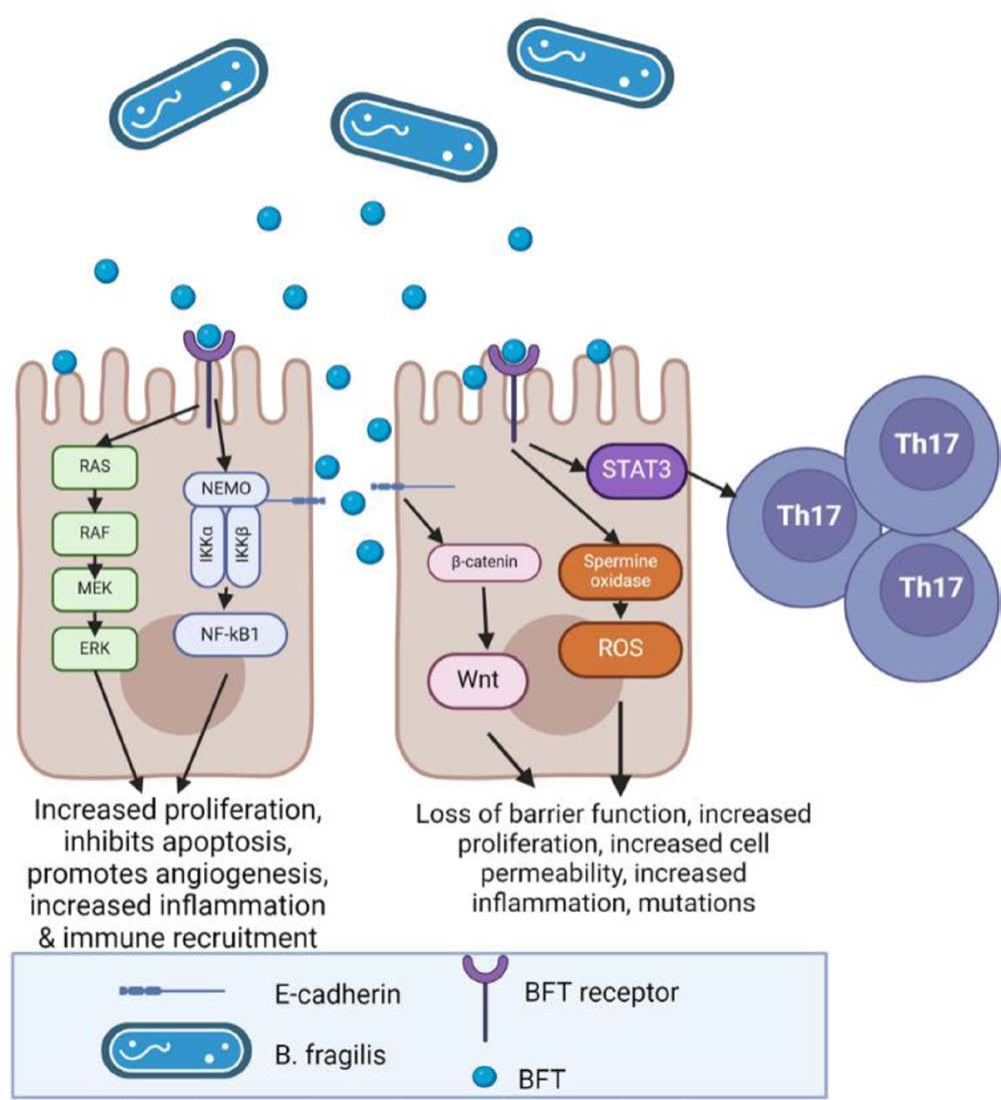
***Bacteroides fragilis*, a member of the microbiome associated with colorectal cancer, produces a metalloprotease toxin, BFT.** This toxin can act in multiple ways. First, BFT can bind to a receptor expressed on epithelial cells, inducing changes in the MAPK and NF-κB pathways. These changes lead to an increase in intestinal epithelial cell proliferation, increased angiogenesis, increased inflammation, and immune recruitment to the area, along with a decrease in apoptosis. These cellular phenotypes are some of the hallmarks of cancer, linking BFT and *B. fragilis* to colorectal cancer. The second method that BFT can affect cells is by acting on the tight junctions between cells. BFT is able to cleave E-cadherin, causing changes in cell signaling via the β-catenin and Wnt pathway. This pathway is highly associated with cancer. Destruction of tight junctions also allows BFT to migrate to the basal side of the epithelial layer and directly interact with immune cells further increases in cell proliferation and metastasis. These interactions lead to a loss in epithelial barrier function and increased cell permeability, triggering an increase in cell proliferation and local inflammation. Additionally, BFT increases spermine oxidase activity in cells, leading to the creation of more reactive oxygen species and increased mutation. Finally, BFT can act on STAT-3, which in turn triggers T_H_17 development. Figures created with BioRender.com (https://www.biorender.com/). BFT, bacteroides fragilis toxin; MAPK, mitogen activated protein kinase; NF-κB, nuclear factor kappa-B.

*B. fragilis* (Figure 2) is an extremely impactful microbe to focus on in CRC development, as it is highly prevalent in samples from CRC patients ^[33]^. The most highly explored way that *B. fragilis* is linked to CRC involves a genotoxin that introduces instability into DNA. While *B. fragilis* is typically a commensal, enterotoxigenic *B. fragilis*, the type that produces bacteroides fragilis toxin (BFT), or *B. fragilis* toxin, is strongly associated with CRC ^[61]^. In susceptible mouse models, enterotoxic *B. fragilis* is able to directly promote tumor formation, while nonenterotoxic *B. fragilis* cannot, directly implicating BFT in CRC causation ^[62]^. Enterotoxic *B. fragilis* was found in 90% of CRC samples, while it was only present in approximately 50% of matched healthy samples ^[63]^. It was also detected in increased quantities in precancerous adenomas and was most strongly associated with earlier stages of CRC ^[64,65]^. Additional correlations were seen between the amount of *B. fragilis* recovered from colon polyps and inflammatory cytokines in the surrounding mucosa ^[33]^. *B. fragilis* toxin is a metalloprotease. This metalloprotease cleaves E-cadherin, a tumor suppressor, increasing colonic epithelial cell proliferation and cell permeability, further increasing inflammation ^[66]^. In addition to actions on E-cadherin, the *B. fragilis* toxin causes activation of the NF-kB and MAPK pathways, leading to epithelial cell proliferation, inhibition of apoptosis, and increased local angiogenesis, all hallmarks of cancer ^[67]^. BFT may help to control which path a tumor develops through, as it is much more highly associated with the serrated CRC developmental pathway as compared to the adenomatous pathway ^[64]^.

Enterotoxigenic *B. fragilis* also impacts the immune system, as exposure to BFT increases interleukin-8 (IL-8) production, causing neutrophil recruitment ^[68]^. BFT is also capable of recruiting neutrophils, and monocytes, as treatment with BFT caused increased expression of the adhesion molecule intercellular adhesion molecule-1 (ICAM-1) ^[69]^. Enterotoxigenic *B. fragilis* is associated with increased STAT-3 signaling, leading to a T_H_17 phenotype and IL-17 production, producing an inflammatory colonic environment ^[63,70,71]^. Incidentally, *B. fragilis* is often also associated with long-term intestinal inflammation seen in IBD ^[72]^.

### 8.2 Streptococcus gallolyticus

*S. gallolyticus* can be detected in up to 50% of CRC tissue samples, while typically present at a level of only 5% in control samples ^[73]^. Previous major *S. gallolyticus* infection resulting in endocarditis or bacteremia has been linked to up to 70% of the CRC cases where this association was investigated ^[74,75]^. Various studies have shown *S. gallolyticus* is associated with increased intestinal inflammation, elevated angiogenesis, heightened cell proliferation, and decreased cell death ^[40]^. These outcomes are potentially due to increased expression of interferon gamma (IFN-γ), cyclooxygenase-2 (COX-2), interleukin-1 (IL-1), IL-8, MYC proto-oncogene (c-MYC), and B-cell leukemia/lymphoma-2 (BCL-2), but the initiation mechanism behind these processes are still unknown ^[73]^. There is, however, a potential link to altered β-catenin signaling, as cells co-cultured with *S. gallolyticus* showed elevated levels of β-catenin ^[76]^. When β-catenin was knocked down, *S. gallolyticus* was no longer associated with increased cell proliferation ^[76]^. The relevance of collagen in these processes has also been explored. When CRC cells were cultured with *S. gallolyticus,* multiple types of collagen were significantly elevated. When either of the two genes for collagen VI production was knocked down, β-catenin signaling and cell proliferation were reduced ^[77]^. Additionally, some studies using various colonic cell lines have seen increased cell proliferation when exposed to *S. gallolyticus* ^[76]^. There is, however, still major work to be done to identify the actual mechanism linking *S. gallolyticus* to CRC. One potential avenue for *S. gallolyticus* to affect CRC is localized binding at or near tumors, followed by signaling alterations that result in changes to cell proliferation rates. One of *S. gallolyticus’* pili, Pil3, efficiently binds to mucins, which are present in the colonic mucosal layer ^[78]^. This binding is especially effective in binding to MUC5AC, which is not typically present in human colons but can be found in nearly 50% of large adenomatous colonic polyps ^[79]^. This pili-mucin binding may bring *S. gallolyticus* into close association with colonic epithelial cells, allowing the bacteria to produce its effects that link *S. gallolyticus* with CRC.

### 8.3 Fusobacterium nucleatum

**Figure 3.**
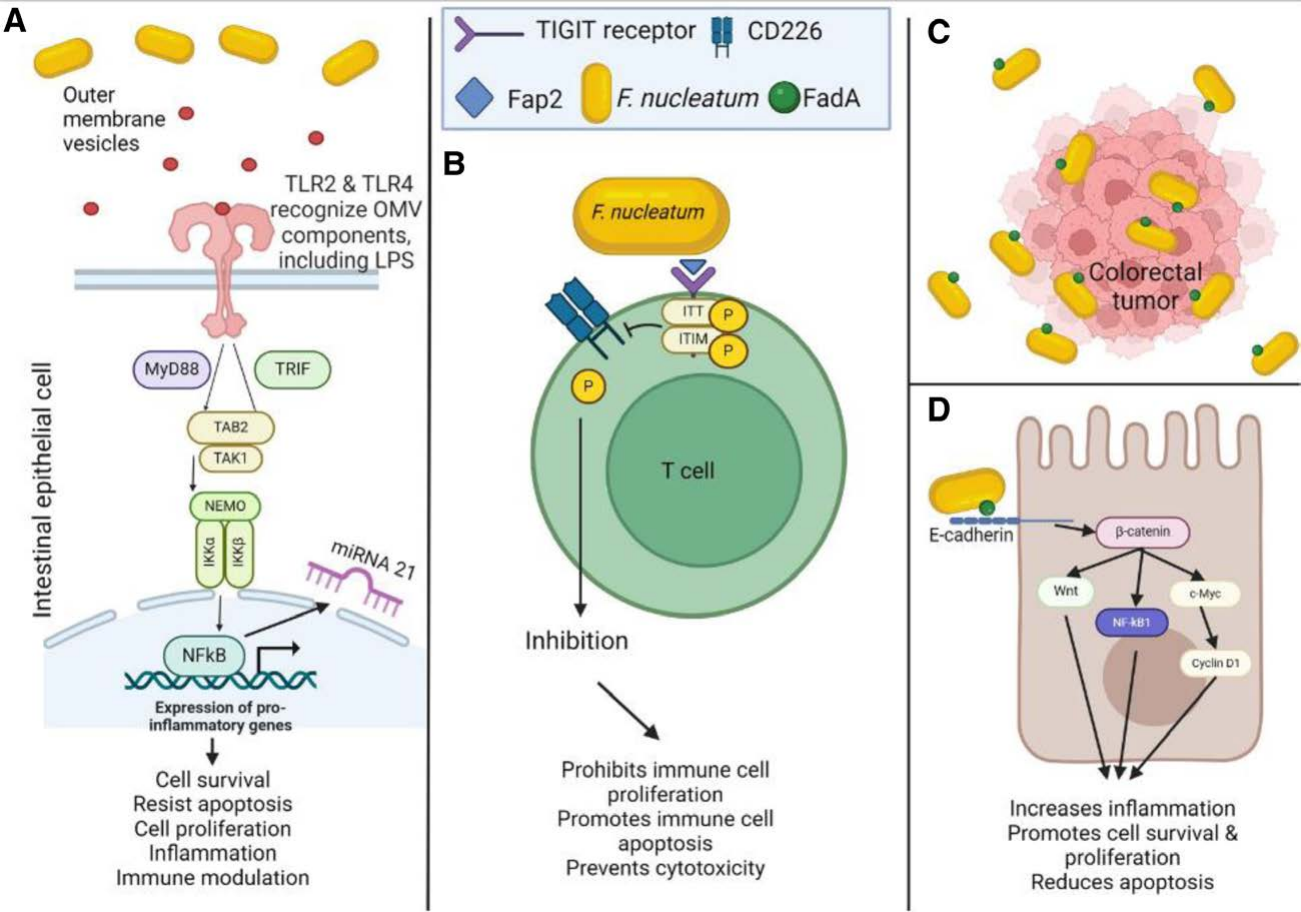
***Fusobacterium nucleatum* is able to act on the immune system. (A**) Components of the bacteria are shed in outer membrane vesicles, which can then be recognized by TLRs present on the surface of cells. Dimer formation then begins the signaling cascade through MyD88 and TRIF, which culminates in NFκB activation and further downstream gene expression. One example of this is increased miRNA 21 expression, culminating in further alterations in gene expression. This results in increased cell survival and proliferation, increased inflammation, decreased apoptosis, and immune changes. (**B**) *F. nucleatum* is also able to directly affect T cells and NK cells, reducing their abilities to recognize and react to immune upsets. This includes the binding of the outer surface protein Fap2 to the TIGIT receptor on T cells. This binding causes the ITT and ITIM motifs to become phosphorylated, which then induces dephosphorylation of CD226, inhibiting T cell activity. This reduces the total number of immune cells available and reduces their ability to exert cytotoxic effects on both microbes and cancer cells. (**C**) *F. nucleatum* also expresses Fap2. Fap2 is frequently used to bind to tumor cells, creating a close association between the bacterium and the cell, classifying *F. nucleatum* as a member of the tumor microbiome. (**D**) Fap2 is able to bind E-cadherin. This results in the release of β-catenin, followed by upregulation of various cell signaling pathways and transcription factors, including Wnt, NF-κB, and c-MYC. These pathways result in increased cell survival and proliferation, a reduction in apoptosis, and promote an inflammatory microenvironment. Figures created with BioRender.com (https://www.biorender.com/). c-MYC, MYC proto-oncogene; ITIM, immunoreceptor tyrosine-based inhibitory motif; ITT, immunoglobulin tail tyrosine; NF-κB, nuclear factor kappa-B; NK, natural killer; TIGIT, T-cell immunoreceptor with immunoglobulin and tyrosine-based inhibitory motif domains; TLR, toll-like receptors.

*F. nucleatum* was first identified as a possible contributor to CRC pathogenesis in 2012 ^[80,81]^. *F. nucleatum* is typically associated with the oral cavity, and when it is introduced to the GI tract may have a role in inducing CRC. In one study, *F. nucleatum* was present in tumor samples in excess of 400 times greater than that which was found in matched control samples and could even be detected inside colonic epithelial cells ^[81]^. Additionally, *F. nucleatum* has been found to cause inflammation, helping to create an environment that is more likely to develop cancer (Figure 3). *F. nucleatum* can activate toll-like receptors (TLR)-2 and TLR-4, potentially through the actions of a group of almost 50 associated miRNAs and the shedding of outer membrane vesicles, activating the NF-kB pathway and producing inflammatory cytokines IL-8, IL-6, and IL-1β ^[82,83]^. Elevated amounts of miRNA 21 are also produced from the activation of this pathway, resulting in further increases in cell proliferation and metastasis and downregulation of apoptosis ^[84]^. *F. nucleatum* can also affect the immune system to reduce the immune response to a tumor. It does this by promoting a proinflammatory state and using its outer surface adhesin protein Fap2 to bind to immune inhibiting receptors, most commonly TIGIT (T-cell immunoreceptor with immunoglobulin and tyrosine-based inhibitory motif domains), on natural killer (NK) and T cells ^[85,86]^. Fap2 has been seen to inhibit the proliferation of T cells, prevent NK cells from effecting their cytotoxic effects on cells, and promote apoptosis of lymphocytes ^[40]^. FadA, another outer surface protein of *F. nucleatum*, acts more like *B. fragilis* toxin, binding E-cadherin and activating β-catenin to promote tumor formation ^[62]^. This action also activates the nuclear factor kappa-B (NF-κB) and c-MYC pathways, further increasing inflammation in the area ^[87]^. Not only are *F. nucleatum* surface proteins important effectors of CRC but so are various metabolites produced by the bacteria. Formate and butyrate are two metabolites known to be produced by *F. nucleatum.* Formate has been shown to increase invasion and stemness of CRC cells, and can also have downstream actions on the Wnt/β-catenin pathway ^[88]^. The effects of formate were not limited to epithelial cell signaling, as exposure to formate also expanded T_H_17 and NK cells in the intestines of mice ^[89]^. Butyrate, another common metabolite produced by *F. nucleatum* and other bacteria, has further been shown to help modulate T_H_17 populations and their associated cytokines ^[90]^. *F. nucleatum* may also have roles in CRC metastasis, as it has been identified in CRC metastases to the liver ^[91]^. Finally, *F. nucleatum* is able to directly bind tumor cells, making it an important member of the tumor microbiome ^[92]^.

### 8.4 *Polyketide synthase +* E*scherichia* coli

Certain *E. coli* strains containing the polyketide synthase (pks) island can produce colibactin, a genotoxin that induces double-stranded breaks in DNA ^[93]^. Nougayrède and colleagues ^[93]^ demonstrated that pks + *E. coli* synthesizes colibactin by the nonribosomal peptide synthetase-pks (NRPS-pks) pathway, which includes a total of 19 genes and is encoded within the 54-kilobase genomic island. Colibactin results in both transient and longer-term DNA damage that is dose-dependent without inducing excessive amounts of cell death ^[94]^. After 72 hours, up to 40% of cells that were exposed to pks + *E. coli* still showed evidence of DNA damage, including chromatin bridges, micronuclei, ring chromosomes, aneuploidy, and polyploidy ^[94]^. Exposure to these *E. coli* strains is linked to increased mutations in the *adenomatous polyposis coli* gene, a known driver mutation site for CRC development ^[95]^. These mutations are frequently single base pair substitutions involving adenine and thymine and are often attributed to pks + *E. coli* exposure at a young age ^[62]^. Studies in mice have directly shown colibactin can induce carcinogenesis, and additional support implicating *E. coli* in CRC development comes from studies showing an association with increased cellular proliferation, induction of mutations, and higher rates of genomic instability ^[40]^. Colibactin may also be responsible for cell cycle arrest in the G2 phase. Studies have shown that colibactin, and thus pks + *E. coli* presence, increases as CRC becomes more advanced ^[65]^. Like other CRC-related bacteria, pks + *E. coli* is also found at an increased rate in IBD patients, although not to the degree in CRC patients ^[62]^.

## 9. Microbiota-immune interactions in cancer—molecular mechanisms

The immune system can either suppress or promote tumor formation, which makes its regulation critical in the progression of CRC. One such example is the dysregulation of cytokine networks, a process central to CRC development through chronic inflammation and tumor microenvironment remodeling ^[96,97]^. Certain microbiota and their metabolic products can disrupt this balance by activating proinflammatory cytokines, chemokines, and their respective networks, setting the stage for CRC development. Here, we will highlight some of the effects on the immune system by the microbiota, including the stages of pathogen recognition, effector molecule production, and effects on immune cells. The intestinal epithelial cells act as a barrier separating both microbiota and pathogenic bacteria from the immune system, but in the process of CRC development, alterations can lead to direct interactions of intestinal bacteria and the wider immune system.

One of the most well-studied interactions of the microbiota and the immune system in the development of CRC is through TLRs). TLRs are key mediators in inflammatory pathways, and their roles in inflammation-driven carcinogenesis, particularly in colitis-associated colorectal cancer (CAC), are being elucidated ^[98]^. TLRs recognize pathogen-associated molecular proteins (PAMPs) and act upstream of immune activators NF-κB, type 1 interferon, and other proinflammatory cytokines ^[99]^. TLRs in the gut are highly compartmentalized, with TLR-2, TLR-4, and TLR-5 being the most prominently expressed TLRs in colonic enterocytes ^[100,101]^. Aberrant regulation of these TLRs could lead to constant activation of the NF-κB signaling pathway, subsequently upregulating proinflammatory cytokines and resulting in a chronic inflammatory environment that is more favorable for CRC and CAC development ^[102]^.

TLR-2 is one of the TLRs expressed in the colon that may have a role in CRC due to its association with inflammatory pathways. When tightly regulated, TLR-2 promotes gut homeostasis by recognizing a variety of components in pathogenic and commensal gram-positive bacteria, including extracellular vesicles ^[103,104]^. However, persistent stimulation of TLR-2 by microbes or microbial products may trigger multiple proinflammatory pathways through the TLR-2/MyD88/NF-κB signaling pathway, setting the stage for chronic inflammation and creating a CAC-conducive environment ^[105]^. After identifying higher TLR-2 levels in CRC tissue relative to healthy tissue, TLR-2’s role was explored via CAC and sporadic CRC mice models ^[106]^. When TLR-2 was knocked out, the number and size of tumors decreased in both models, as did the expression levels of p-NF-κB ^[107]^. The possible role of TLR-2-mediated NF-κB activation in CRC cell growth, invasion, and migration has also been demonstrated in human CRC cell lines ^[108]^. TLR-2 has some inflammatory characteristics that may contribute to CAC, but it is important to note that TLR-2 independent pathways may contribute to this environment and that the specific role of TLR-2 in CRC remains to be determined ^[109]^.

TLR-4 is perhaps the most well-understood TLR in CRC development, with several studies linking microbial activation of TLR-4 to CRC. TLR-4 recognizes a variety of gram-negative bacterial ligands, as well as viruses and fungi ^[110]^. Sustained TLR-4 signaling can increase the risk of CRC by promoting chronic inflammation. Accordingly, TLR-4 has been found to be overexpressed in both human and murine inflammation-associated colorectal neoplasias ^[111]^. TLR-4 promotes CAC development through the upregulation of COX-2, which leads to increased levels of epidermal growth factor (EGFR) signaling ^[111]^. Another study showed that *F. nucleatum* activates TLR-4, which ultimately results in s1000A9 expression and M2 macrophage polarization to help tumor cells achieve immune evasion ^[112]^. Lipopolysaccharide (LPS)-induced TLR-4 activation is linked to CRC recurrence and liver metastasis in CRC cells via phosphoinositide-3 kinase/protein kinase B (PI3K/AKT) signaling, which promotes cancer growth and migration via activation of β1 integrin ^[113]^. There are also indirect effects of *E. coli* promoting CRC invasion and metastasis through cathepsin-K secretion ^[114]^. In this instance, secreted cathepsin-K can bind to TLR-4 and stimulate tumor-associated macrophages to adopt M2 polarization and secrete IL-10 and IL-17, promoting invasion and metastasis ^[114]^. These links indicate that colonic microbiota could activate proinflammatory and proliferative pathways via persistent TLR-4 activation, creating an environment favorable for CRC and CAC development, persistence, and metastasis.

There are many different steps that can occur in the transition from bacterial recognition by the immune system to effector molecule production and immune responses. These different occurrences can influence CRC, and one such pathway is inflammasome activation. Microbiota activation of the inflammasome helps induce proinflammatory signals associated with CRC development. The inflammasome, a multiprotein complex, cleaves proinflammatory cytokines into their active forms upon sensing damage-associated molecular patterns and PAMPs, including both pathogenic and commensal microbes ^[115]^. Inflammasome activation can lead to inflammation, immune cell recruitment, and pyroptosis. When operating in an optimal state, the inflammasome exhibits antitumorigenic properties in both CRC and CAC by downregulating the chemokine chemokine ligand-5 (CCL-5), which in turn downregulates the tumorigenic and inflammatory IL-6 cytokine ^[116,117]^. However, during dysbiosis, typical inflammasome signaling can get disrupted. As a result of this disrupted signaling, *B. vulgatus* and *B. stercoris* can promote CCL-5-mediated IL-6 upregulation, resulting in increased inflammation and tumorigenesis in the colon ^[118]^. Understanding the mechanisms between the microbiome and the inflammasome may provide valuable insights into the development of preventative strategies for CRC.

The gut microbiome can also affect cytokine production. The microbiome has been shown to alter proinflammatory IL-17 production, which appears to accelerate the progression of CRC by interacting with cells in the tumor microenvironment to promote proliferation through the extracellular signal-regulated kinase (ERK), p38 MAPK, and NF-κB signaling pathways, and through inhibition of CD8^+^ and regulatory T cells ^[119,120]^. *E. coli* and *Enterobacteriaceae* drive IL-17C upregulation through TLR-MyD88 signaling in intestinal epithelial cells (IECs), consequently promoting IEC survival in early tumorigenesis through the upregulation of pro-survival genes BCL-xL and BCL-2 ^[121]^. Microbial products can also accelerate tumor growth by interacting directly with cancerous tissue. In colonic tumors, there is decreased mucus and expression of tight junction proteins, making them more permeable to microbial products, and more prone to TLR-activation, leading to IL-23 and IL-17-mediated tumor growth ^[122]^. However, different models of CRC and IL-17 subtypes (IL-17A-F) have varying effects on CRC, suggesting that IL-17’s effect on CRC is context-dependent. For example, one study found that an *Odoribacter splanchnicus* abundant microbiome can stimulate IL-17A-producing T_H_17 cells, resulting in the promotion of epithelial integrity and protecting the environment from colitis and CRC ^[123]^. The complex and dual nature of IL-17 highlights the need for more research to elucidate its precise roles in CRC development and progression, as well as to uncover the various mechanisms by which the microbiota may influence IL-17 signaling.

IL-22, a key regulator of epithelial homeostasis for many organs along the GI tract, is another cytokine implicated in CRC. In noncolitis-associated conditions, IL-22 promotes mucosal wound healing through STAT-3 activation ^[124,125]^. However, IL-22 is much more prevalent in CRC tissue than in healthy epithelial tissue ^[126]^. Consistent with this observation, IL-22 acts as a mediator in the transition from colitis to cancer by directly driving tumor formation and growth through persistent STAT-3 phosphorylation ^[127,128]^. While there is no consensus on the link between IL-22, CRC, and the microbiota, there are several studies showing that microbiota can influence IL-22 expression. Both pathogenic and commensal bacteria upregulate IL-22 expression via myeloid dendritic cells, and IL-22 in turn regulates the composition of the microbiota ^[129,130]^. Additionally, short-chain fatty acids promote IL-22 production by innate lymphoid cells ^[131]^. Although extensive research has been conducted on IL-22-microbial interactions within the context of homeostasis, the precise mechanisms between IL-22, the microbiota, and CRC are yet to be fully understood. However, ongoing research aims to unravel the complex relationship between IL-22, microbiota, and CRC progression.

## 10. Microbiota-immunometabolism interactions in colorectal cancer

Microbial metabolism in the tumor microenvironment affects not only cytokine production but also immune cells in and around the tumor area. Typically, immune cells would recognize and destroy cancer cells, so for cancer to progress into a tumor, something must have gone wrong in this identification and destruction process. In CAC, long-term inflammation from IBD is one of the driving factors of cancer development. The transition from an immune-activated state to an immunosuppressive state is a necessary shift in cancer development, relying on metabolic reprogramming in both tumor cells and immune cells. In CRC, an initial inflammatory state often presents with increases in IFN-γ and other proinflammatory cytokines, as well as increased NF-κB and MAPK transcription due to recognition of PAMPs via TLRs ^[132]^. There are many ways that each of the immune cell types undergo changes during CRC, frequently as a result of microbiota and their metabolites.

Neutrophils are thought to have roles in CRC cell proliferation and immunosuppression, although some studies have noted a possible beneficial effect from neutrophils in the case of early-stage cancers. In contributing to the proinflammatory state of CRC, neutrophils are known to produce IL-23 and IL-1β ^[133]^. Additionally, neutrophils were seen to secrete the chemokine CCL-17, which is a chemoattractant for immunosuppressive Treg cells ^[134]^. Apoptotic CRC cells then go on to excrete IL-8, CXCR (chemokine receptor) 1, and CXCR5, all of which recruit more neutrophils to the area, further compounding the issue of a locally inflammatory and immunosuppressive environment ^[133]^.

In CRC, tumor-associated NK cells are associated with improved survival ^[135]^. Typically, however, CRC-associated NK cells show characteristic differences in comparison to non-CRC-associated NK cells, including decreased activation binding sites and increased inhibitory binding sites ^[136]^. This also results in functional alterations in this NK cell subset. CRC-associated NK cells may become extremely important when looking at metastasis. Liver and lung-resident NK cells are associated with increased survival in CRC patients with metastases to their respective organs. This process is thought to be controlled by IL-18 and IL-1R8 production ^[135]^. NK cell exhaustion was also correlated with shorter survival, resembling what occurs in T and B cell exhaustion ^[137]^.

Different subsets of T cell populations are associated with different CRC groupings but can include T_H_1, T_H_17, Tregs, naïve populations, and cytotoxic T lymphocytes (CTLs). Not only will the types of T cells themselves differ, but so will the location of the T cells within and in the area immediately surrounding the tumor ^[138]^. Just like the other immune cell types, T cells can be associated with certain CRC outcomes ^[139]^. T_H_1 cells are linked to the induction of apoptosis of cancer cells, while T_H_17 cells are associated with increased inflammation ^[138]^. Each subtype has its own unique set of activating molecules and effector cytokines, and each subset will have its own set of effects on CRC as well.

## 11. Applicability to diagnosis, treatment, and prevention

The gut microbiome affects nearly every stage of CRC development—initial carcinogenesis, tumor progression, metastasis, response to treatment, and immune response to the tumor. Harnessing the information the microbiota gives us to be applicable to CRC screening, diagnosis, prognosis, and treatment can only improve our response to CRC moving forward. Microbiome modulation could be one way to use the microbiome to treat or prevent CRC in the future, but to apply the power of microbes purposefully directed against disease states, we must first understand the complexity of interactions occurring in both healthy and CRC states.

In some ways, we can already begin to apply knowledge of the microbiome to CRC. Chemotherapy is one of the most commonly employed treatments for many types of cancer, not just CRC, and microbes have effects on various aspects of chemotherapy. Chemotherapeutic agents typically act indiscriminately on rapidly-dividing cells, and the GI tract is already in a constant state of cell renewal. Therefore, many side effects of chemotherapy affect the GI system, for example diarrhea. Probiotics significantly reduced the incidence of diarrhea in patients undergoing chemotherapy treatment in a meta-analysis of 12 studies, including patients with CRC ^[140]^. The same premise stands true for the prevention and treatment of radiation-induced diarrhea, which occurs in up to 80% of patients receiving radiation to the abdomen or pelvis ^[56]^. However, there is a huge amount of diversity regarding probiotics, including species composition, treatment length, and dose, and so some probiotic treatments could be much more effective than others, and more research needs to be done to establish the most effective ways to use probiotics as a supplemental therapy in the cancer space. Additionally, specific microbial states can be used in predicting the response to various chemotherapeutic regimens. The microbiota can actually be responsible for the metabolism of various drugs used to treat cancer ^[141,142]^. This phenomenon is seen in the breakdown of gemcitabine, a chemotherapeutic drug that works by interfering with DNA synthesis by inhibiting ribonucleotide reductase ^[143]^. Several different typical resident species of the gut microbiome are capable of metabolizing gemcitabine into an inactive form via cytidine deaminase activity, contributing to resistance to the treatment ^[143]^. Approximately ¾ of tumors that were tested for bacteria with the ability to break down gemcitabine contained these bacteria; they also found that co-administration of antibiotics was sufficient to restore the function of gemcitabine ^[143]^. Another case of the microbiome affecting chemotherapy treatment occurs in irinotecan treatment. Here, bacterial β-glucuronidases convert the drug into a toxic byproduct, leading to severe diarrhea as a side effect of irinotecan administration, occurring in up to 80% of patients, with nearly 50% of these cases being classified as severe diarrhea ^[144,145]^.

While traditional cancer treatments such as chemotherapy and surgical removal of tumors are effective ways to limit the progression and spread of cancer, sustained remission is often difficult due to cancer’s ability to evade the immune system. Combined with traditional cancer treatments, immunotherapies are emerging as viable therapeutics to treat CRC by harnessing the immune system’s ability to selectively target cancerous cells, offering the potential for sustained remission. However, the successful application of immunotherapies poses many obstacles and considerations, with the microbiota being an important factor in the ultimate efficacy, efficiency, and toxicity of these treatments. While there are many microbiota-immunotherapeutic interactions, two of the most prominent interactions in this context are the effect of microbiota on immune checkpoint blockade therapies and microbiota interactions in CpG-oligonucleotide immunotherapy.

In cases where immunotherapy is used for cancer treatment, the microbiome may also play a predictive role in response rate. When immunotherapy was used for the treatment of melanoma, specific microbial states were coordinated with therapy response ^[146]^. Patients who responded to the therapy had a higher overall diversity score, coordinating with length of survival. Responders tended to have higher levels of Clostridiales, Faecalibacterium, and Ruminococcaceae, while nonresponders had elevated levels of Bacteroidales and *E. coli* ^[146]^. Additional studies implicated *Akkermansia muciniphilia* and *Roseburia* species as potential biomarkers for response to immunotherapy, as they were coordinated not only with overall response but also with progression-free survival ^[147–149]^. These relationships are present in CRC and in a variety of other types of cancers. In studies including lung cancer, kidney cancer, and melanoma, higher levels of *A. muciniphilia* were coordinated with a better response to PD-1 blockade ^[148]^. Similar results have been observed in survival after chimeric antigen receptor-T cell (CAR-T) cell therapy in lymphoma patients ^[150]^. These microbial states could be extremely important for predicting which patients will respond to immunotherapy, and thus be extremely impactful in treatment decisions for a wide variety of cancers.

In CRC, tumor cells escape immune surveillance in a variety of different ways, one being through immune checkpoint evasion, which typically prevents the immune system from attacking healthy cells. An emerging strategy to combat tumor immune evasion is through immune checkpoint blockade (ICB) therapies, which reverse this evasion mechanism by inhibiting checkpoint components on T cells, but microbial products affect both the efficacy and safety of this therapy ^[151]^. Short-chain fatty acids limit the upregulation of both CD80/CD86 in dendritic cells and inducible costimulators in T cells, increasing the resistance of CRC to cytotoxic T-lymphocyte antigen (CTLA)-2 blockade therapy ^[152]^. Different combinations of ICB treatment appear to increase clinical benefit—for example, CTLA-4 blockade therapy has been shown to have some clinical benefit when combined with PD-L1 blockade in CRC treatment ^[153]^. In this combined therapy approach, a higher abundance of *B. intestinalis* was associated with more immune-related adverse events during ICB treatment ^[154]^. Given the complex relationship between microbiota and ICB, current research seeks to better understand which microbiota increase the resistance and toxicity of ICB therapies.

Microbes can also enhance the antitumor effect of immunotherapies. *B. thetaiotaomicron* and *B. fragilis* were associated with increased efficacy to CTLA-4 blockade in sarcomas by stimulating T_H_1 cell activation and promoting the maturation of dendritic cells ^[155]^. While the *Bacteroides* species have previously been associated with CRC, this finding highlights the complexity of microbial interactions in different stages and contexts of CRC development. Additionally, when anti-PD-1 therapy was combined with oral administration of *Bifidobacterium*, it nearly abolished melanoma tumor growth by increasing dendritic cell function, leading to enhanced CD8^+^ T cell priming and accumulation at the tumor microenvironment ^[156]^. Furthermore, *Lactobacillus rhamnosus* GG augmented the antitumor response in ICB therapy by triggering dendritic cells to produce type I interferon and enhancing cross-priming of antitumor CD8^+^ T cells ^[157]^. The many diverse and complex ways that microbiota can increase or decrease ICB’s effects in CRC and other cancers highlight the importance of considering microbiota composition in ICB application.

Another immunotherapy used specifically for CRC treatment is CpG-oligonucleotide immunotherapy, which is an immunostimulatory approach usually used in combination with other therapies to treat CRC ^[158]^. In this therapy, prokaryotic DNA is delivered to the tumor to activate the innate and adaptive immune responses, resulting in an antitumor immune response. However, antibiotic treatment simulating gut dysbiosis diminishes the effect of CpG-oligonucleotide immunotherapy by lowering cytokine production and tumor necrosis factor (TNF) expression ^[159]^. This diminishing effect is dependent on which genera of bacteria are present in the gut, with *Alistipes* and *Ruminococcus* increasing TNF production and treatment efficacy, and *Lactobacillus* decreasing TNF production, ultimately affecting how well myeloid-derived cells responded to therapy ^[159]^. This suggests that the efficacy of CpG treatment is at least partially dependent on the composition of the microbiome.

When we understand how the microbiome affects CRC, the potential exists to use microbial modulation principles to improve CRC outcomes. Fecal microbiota transplants have been used extremely successfully for recurrent *Clostridioides difficile* infections and are being tested for use in a much wider variety of diseases ^[160–163]^. Several mouse studies showed fecal microbiota transplants from mice that responded to immunotherapy treatment to nonresponsive mice allowed the previously nonresponsive mice to gain a therapeutic response, and human clinical trials are underway for a number of related projects ^[164]^. In addition to direct microbial therapy, the microbiome can be used in many other manners—to alleviate symptoms resulting from chemotherapy and radiation, to predict and improve outcomes of various immunotherapies in a variety of cancers, to stratify risk groups for CRC screening, and to reduce the risk of developing CRC, especially in already high-risk groups. These applications demonstrate the enormity of potential that the microbiome possesses in applicability for improving the survivability of cancer and the experiences of those individuals living with cancer.

These immunotherapy studies identify microbiota as important mediators in the immune response, which is central to immunotherapy efficacy and safety. By understanding how different strains of bacteria induce different immune responses, we can not only better predict the outcome of these therapeutic strategies but also modulate the microbiota composition to maximize immunotherapeutic efficacy and ultimately patient outcomes. In translation, this may mean altering the microbiome composition by dietary and nutrition patterns, taking probiotics, or applying fecal transplants in combination with immunotherapies to optimize outcomes ^[165]^.

## 12. Conclusions

The gut microbiome undoubtedly has impacts on CRC—the “invisible organ” living throughout our bodies does not have an invisible impact on our health. The sheer quantity of microbes, metabolites, and interactions in the intestine mean more research is needed to fully understand the complex set of interactions that occur in a healthy individual or in someone with CRC. We do know, however, that CRC is associated with microbial dysbiosis. Changes in the microbiome also lead to changes in bacterial metabolism. These changes then go on to have effects on other residents of the microbiome, intestinal epithelial cells, and immune cells. The culmination of all these changes and interactions is part of what results in the imbalance in immunometabolism seen in CRC. Here, we have explored some of the key players in CRC from multiple aspects: the microbiome, microbial metabolites, metabolism, and the immune system. How each of these components interact is part of the key to understanding how and why CRC develops, progresses, and metastasizes. By increasing our understanding of these processes, we can create innovative solutions to some of the most complicated problems in medicine. Every day, we are learning more about how the microbiome, cancer metabolism, and the immune system interact to develop new predictive strategies for outcomes and treatment responses, developing complementary therapies to lessen the side effects associated with conventional cancer treatment, and considering how best to develop completely new therapies and prevention strategies for a variety of cancers. These tasks will only lead to improved outcomes and a decreased burden of cancer for all.

## Conflicts of interest

The authors declare no conflicts of interest.

## Funding

This study was supported in part by the National Institute of Health NIDDK. R56DK136728 (A.A.), P20GM130456 (A.A.; Alam Project ID9790), K01DK114391 (A.A.), ACS IRG (A.A.), and Elsa U. Pardee Foundation Grant (A.A.).
